# Poststreptococcal syndrome mimicking conjunctival lymphoma

**DOI:** 10.1186/1471-2334-13-149

**Published:** 2013-03-25

**Authors:** Iwona Rospond-Kubiak, Agata Brązert, Jarosław Kocięcki, Jan Bręborowicz

**Affiliations:** 1 Department of Ophthalmology, Poznań University of Medical Sciences, 1/2 Długa St., Poznań, 61-848, Poland; 2 Department of Oncologic Pathology, Chair of Oncology, Poznań University of Medical Sciences, 1/ 2, Łąkowa St, Poznań, 61-878, Poland

**Keywords:** Postreptococcal syndrome, Anti-streptolysine 0, Conjunctival lymphoma

## Abstract

**Background:**

Poststreptococcal syndrome (PSS) can be a consequence of nonpurulent primary infection with group A streptococci (GAS). Postreptococcal uveitis is a well recognized entity with quite a few descriptions in the literature, but so far no conjunctival involvement has been reported.

The aim of the study is to present a rare case of postreptococcal conjunctival lesions mimicking a lymphoma.

**Case presentation:**

19-years-old Caucasian female presented with pink, nodular infiltrates in the right conjunctiva that occurred a few months after upper respiratory tract infection and tonsillectomy. Histopathological examination of collected lesion samples revealed inflammatory reaction with lymphocytes proliferation and failed to rule out a myeloma. Complementary flow-cytometry did not show monoclonal proliferation of lymphocytes B. During follow-up we observed the complete regression of conjunctival lesions after the benzyl penicillin treatment prescribed by ENT specialist due to elevated plasma ASO levels. Therefore, we suppose that those lesions must have represented a part of poststreptococcal syndrome.

**Conclusions:**

To conclude, this is, to the best of our knowledge, the first report of conjunctival involvement in the course of PSS related to group A streptococci.

## Background

Poststreptococcal syndrome (PSS) is a sterile inflammation thought to represent an autoimmune reaction between streptococcus-sensitized lymphocytes and host tissue because of ‘molecular mimicry’. It can be a consequence of nonpurulent primary infection with group A streptococci (GAS). The most common forms of PSS are: rheumatic fever, acute poststreptococcal glomerulonephritis and erythema nodosum.

Ocular tissues involvement in PSS has been rarely reported in literature in the form of poststreptococcal uveitis secondary to group A streptococcal infection [1-5] and keratouveitis after a pharyngitis caused by group C streptococci (GCS) [6]; but, to date, conjunctival involvement has not been reported.

Poststreptococcal uveitis can affect any part of the uvea and is almost always bilateral, nongranulomatous with the occasional formation of posterior synechiae. It appears to be a disease of young patients (96% under 40 years of age, and more than 50% younger than 15 years) [4]. There is a controversy regarding the recognition of PSS syndrome only after an elevated anti-streptococcal lysine O (ASO) titer, which may variate regarding the patient age, ethnicity and season of the year [3]. The streptozyme agglutination test (SAT) represent another diagnostic option.

The aim of the study is to present a rare case of postreptococcal conjunctival lesions mimicking a lymphoma.

## Case presentation

A 19-year-old Caucasian female was referred to the Ocular Oncology Service, Department of Ophthalmology, Poznań, Poland in April 2009 with a suspicion of conjunctival lymphoma. The patient reported the appearance of painless reddish nodules on the right bulbar conjunctiva couple weeks previously. The patient didn’t have past ocular history of note. Her past medical history revealed history of purulent tonsillitis treated with systemic antibiotic therapy a year earlier. However, after this treatment the levels of antistreptolysin (ASO) were still elevated (up to 500 U/ml) and the patient had undergone tonsillectomy three months later. The working clinical diagnosis by her local ophthalmologist was of suspected lymphoma and the patient received no topical treatment. At presentation to our department, the ASO level was 482 U/ml (N: 10–200 U/ml) and other laboratory test results were within normal limits. The patient was otherwise healthy with no travelling history and reported no coincidence between the appearance of conjunctival lesions and tonsillitis.

**Figure 1:**
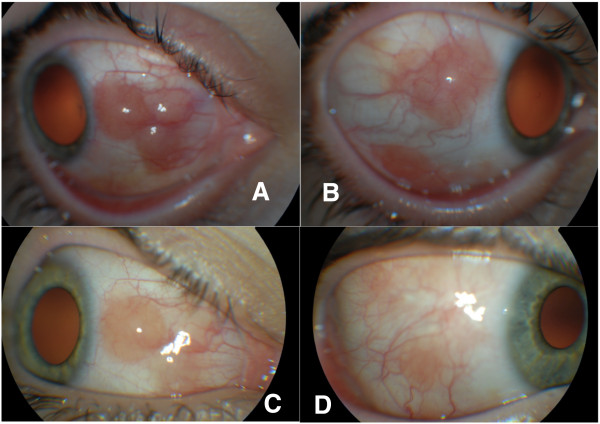
(A + B) - Anterior segment of right eye (RE) – April 2009, and disappearance of conjunctival lesions after benzyl penicilin treatment in June 2009 (C + D).

On ocular examination, the best corrected visual acuity was 5/5 with the right eye and 5/5 with the left eye, which was healthy. On biomicroscopy there were numerous, pink and reddish nodules on the right bulbar conjunctiva nasally and temporally [Figure 1A and B]. Those lesions seemed to be tightly fixed to the sclera. There was a similar subconjunctival mass in the right lower fornix. Otherwise the anterior segment appeared normal, with no signs of uveitis or keratitis. The right fundus appeared normal. Orbital ultrasound revealed no pathology. No lymphadenopathy was noted. Because the observed lesions did not have the typical appearance of a conjunctival lymphoma and the diagnostic uncertainty, incisional biopsy was taken.

**Figure 2:**
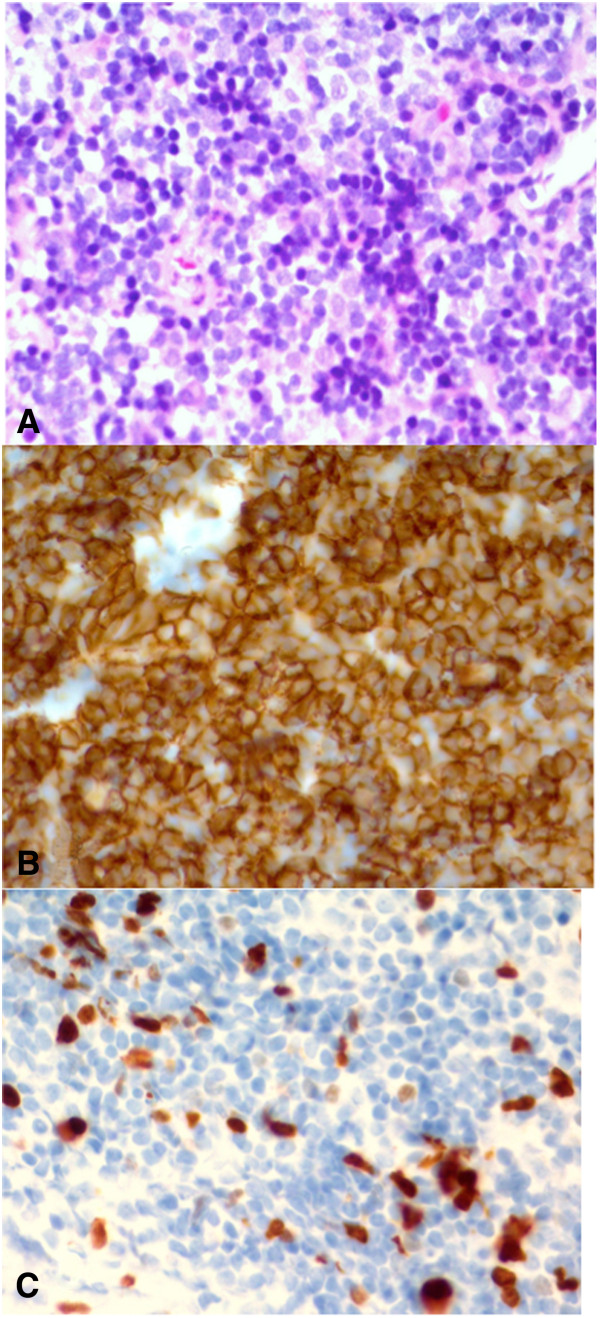
Histopathologic staining of the lesion samples (A) – H + E, (B) – CD 20 (+) and (C) – Ki67

The histopathological assessment of the collected specimens revealed infiltration by plasma cells and lymphocytes. The immunohistochemistry enabled recognition of B cells (CD 20 +) and T cells (CD3 +), which showed enhanced expression of Ki-67. Expression of CD 10, CD 23, CD 43 and bcl2 was also detected [Figure 2]. This might have corresponded to an inflammatory process, but also to a conjunctival infiltration in the course of multiple myeloma. As a part of further management, a bone marrow biopsy was performed. Flow cytometry revealed 25.5% lymphocytes without evidence of monoclonality, 4% monocytes, 69.5% neutrophils and 1% mieloblasts. Other systemic investigations (chest X-ray, abdomen ultrasound) revealed no pathology.

On the next follow-up visit, 2 months later, in June 2009, shrinking and pallor of the conjunctival lesions was noted. [Figure 1C and D]. The patient reported receiving treatment with benzyl penicillin (6×2.4 mln units every two weeks) 4 weeks earlier by the ENT specialist because of persistent elevation of the plasma concentration of ASO. At the next follow-up in 6 weeks time the conjunctival lesions had completely disappeared.

## Discussion

In view of the clinical progress of our case, the likely diagnosis of poststreptococcal syndrome (PSS) affecting the conjunctiva that had responded to penicillin treatment was made. The differential diagnoses for that case would be anterior scleritis or episcleritis, conjunctival lymphoma, ocular infiltration in the course of multiple myeloma, reactive lymphoid hyperplasia, tuberculosis, chlamydia or herpetic infection, papilloma and superficial migratory phlyctenulosis.

The first two conditions could be ruled out since the lesions initially were painless. However, a multifocal nodular episcleritis and scleritis can sometimes appear in the course of Hodgkin’s lymphoma but the onset of a disease in the cases reported in the literature was associated with pain [7]. Similarly, the clinical findings could have been part of myeloma [8]. Although histopathologic examination revealed an infiltration by plasma cells and lymphocytes, further hematologic and systemic investigations (bone marrow biopsy and flow cytometry) ruled out multiple myeloma and other lymphoproliferative disease [9]. There was no discharge, no lymphadenopathy, no corneal involvement at first presentation and no systemic signs of infectious disease which let us to rule out tuberculosis, chlamydia or herpes infection although the collected lesion samples were not evaluated with PCR for a specific DNA.

Furthermore, we observed the diminution of all lesions after benzyl penicilin treatment due to elevated plasma concentration of ASO which seems to confirm the final diagnosis.

## Conclusion

To conclude, this is, to the best of our knowledge, the first report of conjunctival involvement in the course of PSS related to group A streptococci.

Consent

Written informed consent was obtained from the patient for publication of this Case report and any accompanying images. A copy of the written consent is available for review by the Series Editor of this journal

## Competing interests

The authors declare that they have no competing interests.

## Authors’ contributions

IRK performed the incisional biopsy, was responsible for the clinical management of the patient, prepared most of the manuscript; AB collected the photograps and prepared a clinical part in the first draft of the manuscript, JK supervised the clinical management of the patient, made the literature search and set the final diagnosis, JB evaluated the histopathology. All authors revised and accepted the final version of the manuscript.

## Pre-publication history

The pre-publication history for this paper can be accessed here:

http://www.biomedcentral.com/1471-2334/13/149/prepub
